# Vitamin D and Bladder Cancer Risk: An Umbrella Review and Second Order Meta‐Analysis

**DOI:** 10.1002/cam4.71672

**Published:** 2026-03-11

**Authors:** Stefano Mancin, Gaetano Ferrara, Sara Morales Palomares, Sofia Matteucci, Alice Maria Santagostino, Giulia Ferrari, Mauro Parozzi, Fabio Petrelli, Rodolfo Hurle, Marco Sguanci, Giovanni Cangelosi

**Affiliations:** ^1^ IRCCS Humanitas Research Hospital Milan Italy; ^2^ Department: Nefrology and Dialysis Unit Ramazzini Hospital Carpi Modena Italy; ^3^ Department of Pharmacy Health and Nutritional Sciences (DFSSN), University of Calabria Rende Italy; ^4^ Department of Medicine and Surgery University of Parma Via Antonio Gramsci Parma Italy; ^5^ School of Pharmacy, Polo Medicina Sperimentale e Sanità Pubblica “Stefania Scuri” Camerino Italy; ^6^ Italian Society of Nephrology Nurse (SIAN) Olbia Italy; ^7^ Units of Diabetology ASUR Marche Fermo Italy

## Abstract

**Introduction:**

Bladder cancer is a common malignancy with a high recurrence rate, posing a significant public health burden. Beyond its role in bone health, vitamin D has been suggested to influence cancer risk, including bladder cancer.

**Aim:**

To evaluate the association between serum vitamin D levels, dietary intake, and supplementation with bladder cancer risk.

**Methods:**

This umbrella review included systematic reviews and meta‐analyses from multiple databases, focusing on serum levels or intake of vitamin D and bladder cancer risk. Study quality was assessed using the JBI Critical Appraisal Tool. The Corrected Covered Area showed a 15.5% overlap. Considerable heterogeneity in study design, populations, and vitamin D assessment was noted. Two second‐order meta‐analyses were conducted for quantitative synthesis.

**Results:**

Eight studies met the inclusion criteria. Adequate serum vitamin D was generally defined as levels ≥ 30 nmol/L. Low serum vitamin D was significantly associated with increased bladder cancer risk (RR = 1.32; 95% CI: [1.27, 1.38]). Higher serum levels were linked to a non‐significant risk reduction (RR = 0.86; 95% CI: [0.63, 1.16]). Evidence on dietary intake was inconsistent, with some analyses suggesting a modest protective effect, particularly when combined with specific dietary patterns.

**Conclusions:**

Low serum vitamin D is consistently associated with increased bladder cancer risk, while maintaining levels above 30 nmol/L may provide some protection. Evidence on dietary intake and supplementation remains inconclusive. Future studies should adopt standardized methods for vitamin D measurement, explore the role of free versus total vitamin D, and clarify population‐specific differences to better define vitamin D's role in bladder cancer prevention.

## 1 Introduction

Bladder cancer is one of the most prevalent malignancies worldwide, ranking sixth in incidence and ninth in mortality within the global oncology landscape. In 2020, over 573,000 new cases and 212,000 deaths were reported [1]. Most bladder cancer cases occur in high‐income countries, with the highest rates observed in North America and Europe. A distinctive feature of bladder cancer is its high recurrence rate: approximately 65% of patients with non‐invasive or in situ tumors and 73% of those with advanced disease at diagnosis experience a recurrence within 5 years. This makes bladder cancer one of the most burdensome cancers and a significant public health challenge [1, 2, 3]. The complexity of bladder cancer arises not only from genetic predisposition but also from a broad spectrum of environmental and behavioral risk factors. These include cigarette smoking, occupational exposure to toxic chemicals, and male sex. Additional factors, such as alcohol consumption, body mass index (BMI), physical activity, and nutrition, have also been identified as potential contributors to bladder cancer risk [1, 4]. Given the bladder's key role in the excretory process, it is estimated that one‐third of bladder cancer cases could be prevented by adhering to specific dietary recommendations [5].

In this context, recent research has focused on the role of vitamin D and its potential protective effects against cancer, including bladder cancer. Traditionally recognized for its role in maintaining calcium and phosphorus homeostasis and supporting bone mineralization, vitamin D exists in two forms: D2 (ergocalciferol), obtained from diet and supplements, and D3 (cholecalciferol), primarily synthesized in the skin through sunlight exposure. Both forms are initially inactive and require enzymatic transformations in the liver and kidneys to become the active form, calcitriol, which regulates gene expression via the vitamin D receptor [6, 7].

Vitamin D may influence bladder cancer risk through multiple mechanisms, including inhibition of cell proliferation, induction of programmed cell death (apoptosis and autophagy), suppression of angiogenesis, modulation of intracellular calcium signaling, and enhancement of antitumor immune responses. These multifaceted anticancer effects position vitamin D as a promising agent in bladder cancer prevention and management [8, 9, 10, 11, 12, 13, 14, 15, 16, 17, 18, 19, 20, 21]. Low vitamin D levels have been associated with an increased risk and growth of both solid and non‐solid tumors, while supplementation in cancer patients has been linked to more favorable prognoses [10, 11, 12, 13, 14, 15]. Additionally, vitamin D may affect the inflammatory state of the tumor microenvironment and immune cell infiltration, thereby enhancing the immune response [21]. Evidence from epidemiological and experimental studies suggests that using vitamin D in combination therapy can enhance the efficacy of antitumor drugs, such as Gemcitabine, Cisplatin, Doxorubicin, and proton therapy [22, 23, 24]. An emerging aspect is vitamin D's role in counteracting drug resistance induced by chemotherapeutics and targeted therapies, such as tyrosine kinase inhibitors (TKIs) [25, 26]. Although the molecular mechanisms underlying this effect remain under investigation, in vitro studies on bladder cancer indicate that the active form of vitamin D, 1,25(OH)_2_D_3_, may inhibit tumor cell migration and invasion, limiting tumor progression. Moreover, vitamin D has been identified as a key modulator of bladder epithelium integrity, acting as a natural barrier against uncontrolled tumor cell proliferation [19, 20]. Beyond its direct effects, vitamin D may indirectly influence bladder cancer development through its role in calcium and phosphorus metabolism [27]. Adequate calcium availability has been correlated with a protective effect against various types of cancer, including bladder cancer, whereas vitamin D deficiency may impair intestinal calcium absorption, contributing to tumor progression. Alterations in inorganic phosphate levels have also been associated with cellular dysfunctions, potentially linked to the development of neoplasms and other pathological conditions, such as hypertension [28]. Despite the growing body of research, previous meta‐analyses and systematic reviews on the association between vitamin D and bladder cancer risk have shown considerable heterogeneity in study designs, populations, and methods, often focusing separately on either serum vitamin D levels or dietary intake. Furthermore, overlapping primary studies within these reviews may have biased aggregated results. Therefore, this umbrella review aims to comprehensively synthesize existing evidence, integrating findings on both serum vitamin D status and dietary intake, while addressing study overlap and methodological variability to clarify the relationship between vitamin D and bladder cancer risk.

### 1.1 Objective

This umbrella review aimed to assess the association between serum vitamin D levels and the risk of bladder cancer. Additionally, it sought to evaluate the impact of diet and vitamin D supplementation on this risk. Given that serum vitamin D levels and nutritional intake represent related but biologically distinct parameters, where serum levels reflect the actual physiological status of vitamin D in the body, and dietary intake indicates the exogenous sources, this review includes both to provide a comprehensive assessment of vitamin D's role in bladder cancer risk.

## 2 Methods

The protocol was registered in the Open Science Framework (OSF) database and is accessible at the following link: https://doi.org/10.17605/OSF.IO/8HWN3. The methodology adhered to the guidelines outlined in the Joanna Briggs Institute (JBI) [29], which provides comprehensive instructions for conducting umbrella reviews. This umbrella review was reported according to the Preferred Reporting Items for Systematic reviews and Meta‐Analyses (PRISMA) guidelines [30].

### 2.1 Research Question

The research question for this umbrella review was defined as: “What is the association between vitamin D levels and the risk of developing bladder cancer?” This question was formulated following the PICO framework [31], which structures scientific research. The population (P) includes adults at risk for or diagnosed with bladder cancer. The intervention (I) of interest pertains to vitamin D levels, measured by serum 25(OH)D concentration. The comparison (C), where available, was between those with suboptimal vitamin D levels versus those with optimal levels. The outcome (O) analyzed was the incidence of bladder cancer or associated mortality. This review also considers vitamin D intake through diet and supplementation to address the potential differences in biological impact between intake and serum status.

### 2.2 Eligibility Criteria

Only systematic reviews or meta‐analyses published in English and relevant to the research question of this umbrella review were included. Studies with low methodological quality or not available in full text and not meeting the stated inclusion criteria were excluded.

### 2.3 Search Strategy

A comprehensive search was conducted across multiple databases, including PubMed, Cochrane Library, Cumulative Index to Nursing and Allied Health Literature (CINAHL), and Embase, to identify systematic reviews and meta‐analyses examining the association between vitamin D levels and bladder cancer incidence or mortality. Although the primary study objectives initially focused on serum vitamin D, the search strategy was intentionally designed to encompass all relevant forms of vitamin D exposure, including serum levels, dietary intake, and supplementation to ensure a more comprehensive evaluation of the potential relationship between vitamin D and bladder cancer outcomes. No temporal restrictions were applied. The search strategy combined the terms “vitamin D” and “bladder cancer” using the Boolean operator AND. To enhance sensitivity, synonyms and relevant MeSH terms were included for both concepts, such as “urothelial carcinoma”, “transitional cell carcinoma”, “non‐muscle invasive bladder cancer”, and “metastatic bladder cancer” for bladder cancer; and “cholecalciferol”, “ergocalciferol”, “25‐hydroxyvitamin D”, and “1,25‐dihydroxyvitamin D” for vitamin D. These terms were combined using OR operators as appropriate to capture all relevant variations (Table S1). Searches were limited to articles published in English. Reference lists and citations of selected full‐text articles were manually screened to identify additional eligible studies. To ensure broader coverage, supplementary searches were conducted using Google Scholar and Open Gray to capture gray literature. All potentially relevant records were imported into EndNote 20 software (available at: https://endnote.com/). The detailed search strategy for each database is reported in Table S1.

### 2.4 Study Selection

Study selection was carried out using a three‐phase approach. Duplicate records were initially removed using EndNote 20 software, followed by further manual removal to ensure an accurate compilation of the literature corpus [32]. The next steps involved screening titles and abstracts, followed by full‐text evaluation. Two independent reviewers assessed studies for inclusion, with a third researcher consulted in cases of disagreement.

### 2.5 Quality Assessment of Included Studies

The methodological quality of the included systematic reviews was assessed using the JBI Critical Appraisal Tool for Systematic Reviews [29] (see Table S2 for the complete checklist). This tool evaluates critical aspects, including the quality of the bibliographic search, risk of bias in included studies, adequacy of meta‐analytic methods, and the assessment of publication bias. High‐quality studies were identified based on a previous study [33] in which studies with a JBI score higher than 70% were classified as having a high quality, those with a score between 50% and 70% as having a medium quality, and those with a score less than 50% as having a low quality.

### 2.6 Assessment of Overlap

Building on previously published studies [34], an additional analysis was performed to evaluate the overlap of primary studies included in the systematic reviews. A citation matrix was constructed, and the Corrected Covered Area (CCA) [35] was calculated using the GROOVE tool to quantify the degree of overlap [36]. The CCA was interpreted as follows: slight if < 5%, moderate if ≥ 5% and < 10%, high if ≥ 10% and < 15%, and very high if ≥ 15% [35]. This step was essential in identifying the redundancy of primary studies reported in multiple reviews, as shown in Table S3.

To clarify the reasons for the observed overlap, exposure types (serum, diet, supplement) and outcomes were added for each included study in Tables S3 and S4. This addition highlights the methodological heterogeneity across reviews, which may explain the very high overlap observed (CCA = 15.5%).

### 2.7 Data Extraction

Data extracted from each review included the author, publication year, country, study population, exposure variables (serum, diet, supplement), outcomes (bladder cancer incidence or mortality), estimated effects, and heterogeneity assessment, quality assessment. Data extraction was conducted independently by two reviewers, with conflicts resolved through discussion or by a third reviewer.

### 2.8 Data Synthesis

Two second‐order meta‐analyses were performed to quantitatively synthesize the association between vitamin D levels and bladder cancer risk. The first meta‐analysis synthesized results where adequate or high serum vitamin D levels in relation to bladder cancer risk were reported; the second meta‐analysis synthesized, on the other hand, the risk of bladder cancer associated with low serum vitamin D levels (< 30 nmol/L).

Both analyses utilized risk ratios (RR) and their corresponding 95% confidence intervals (95% CI). For each meta‐analysis, both common effect and random effects models were applied. The random effects models included Hartung–Knapp adjustments to provide more accurate estimates in the presence of heterogeneity.

The inverse variance method was employed for effect size pooling, while the between‐study variance (τ^2^) was estimated using the DerSimonian–Laird method. For the first meta‐analysis, confidence intervals for τ^2^ and τ were calculated using the Jackson method. Heterogeneity was quantified with the *I* ^2^ statistic. The random effects models incorporated the Hartung–Knapp adjustment; this adjustment was adopted to provide a more conservative and robust estimate of the confidence intervals, while acknowledging that its validity may be limited in this context. Study weights were calculated separately for the common effect and random effects models.

The first meta‐analysis included four studies and showed substantial heterogeneity (*I* ^2^ = 81.0%), supporting the use of the random effects model. The second meta‐analysis included two studies and did not exhibit evidence of heterogeneity (*I* ^2^ = 0%), resulting in overlapping estimates between the common effect and random effects models. Analyses were performed with R 4.4.2 software and “meta” library package.

## 3 Results

### 3.1 Study Selection

**FIGURE 1:**
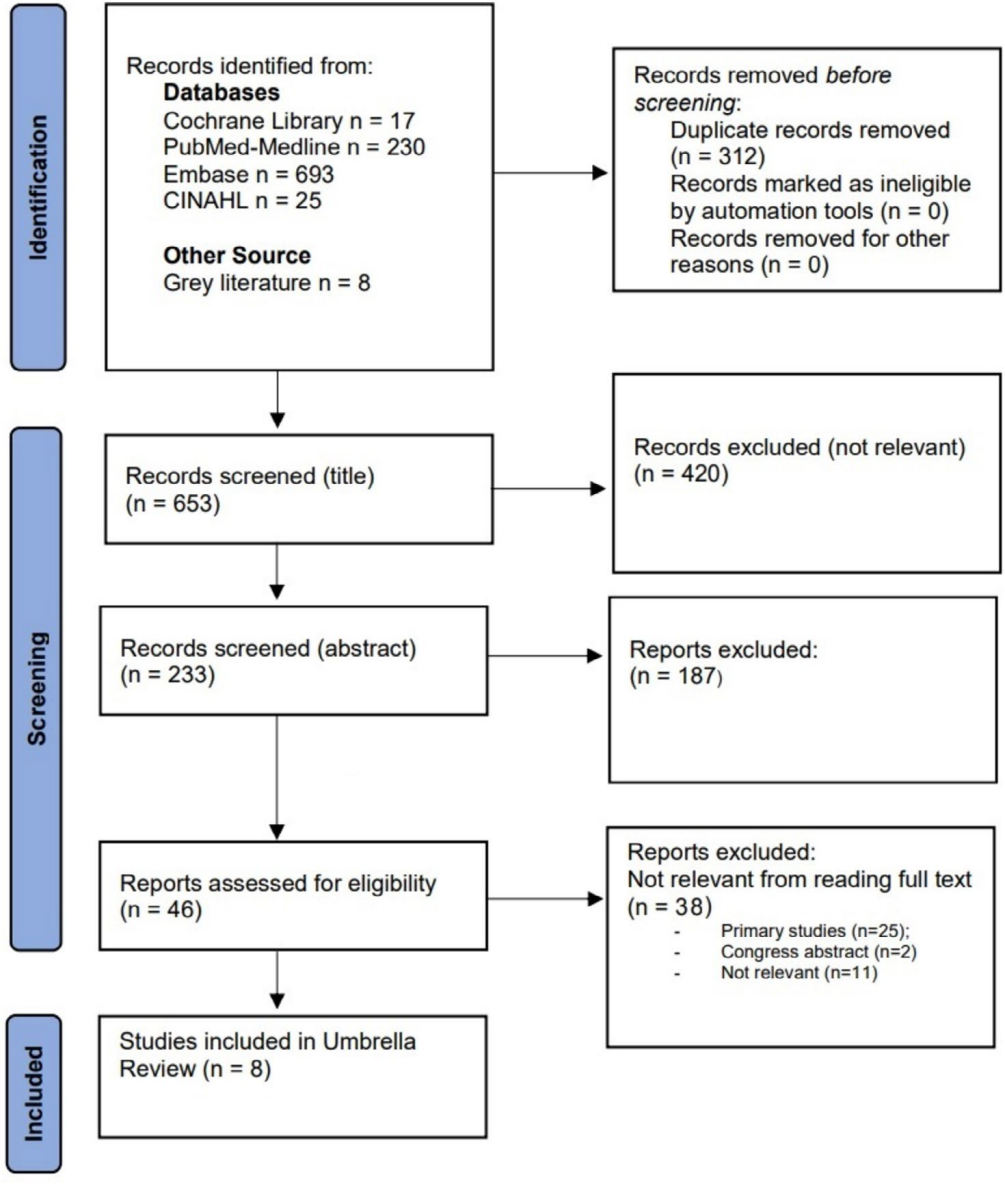
Flow‐chart of study selection. PRISMA flow chart.

A comprehensive search across biomedical databases yielded a total of 973 records: PubMed/Medline (*n* = 230), Embase (*n* = 693), CINAHL (*n* = 25), Cochrane Library (*n* = 17). The search of gray literature revealed eight additional records. Initially, 312 records were excluded due to duplication; the subsequent screening process commenced with a thorough examination of 653 article titles, resulting in the selection of 233 articles deemed pertinent, while 420 were excluded. Subsequently, all abstracts were subjected to analysis, leading to the exclusion of 187 irrelevant articles, while 46 records were considered relevant for a full analysis. Of these, 38 articles were excluded for the following reasons: (a) primary studies (*n* = 25); (b) congress abstract (*n* = 2); (c) not relevant (*n* = 11). Ultimately, following the screening process, eight articles were deemed eligible for inclusion in this umbrella review (Figure 1).

### 3.2 Quality and Bias Assessment

**FIGURE 2:**
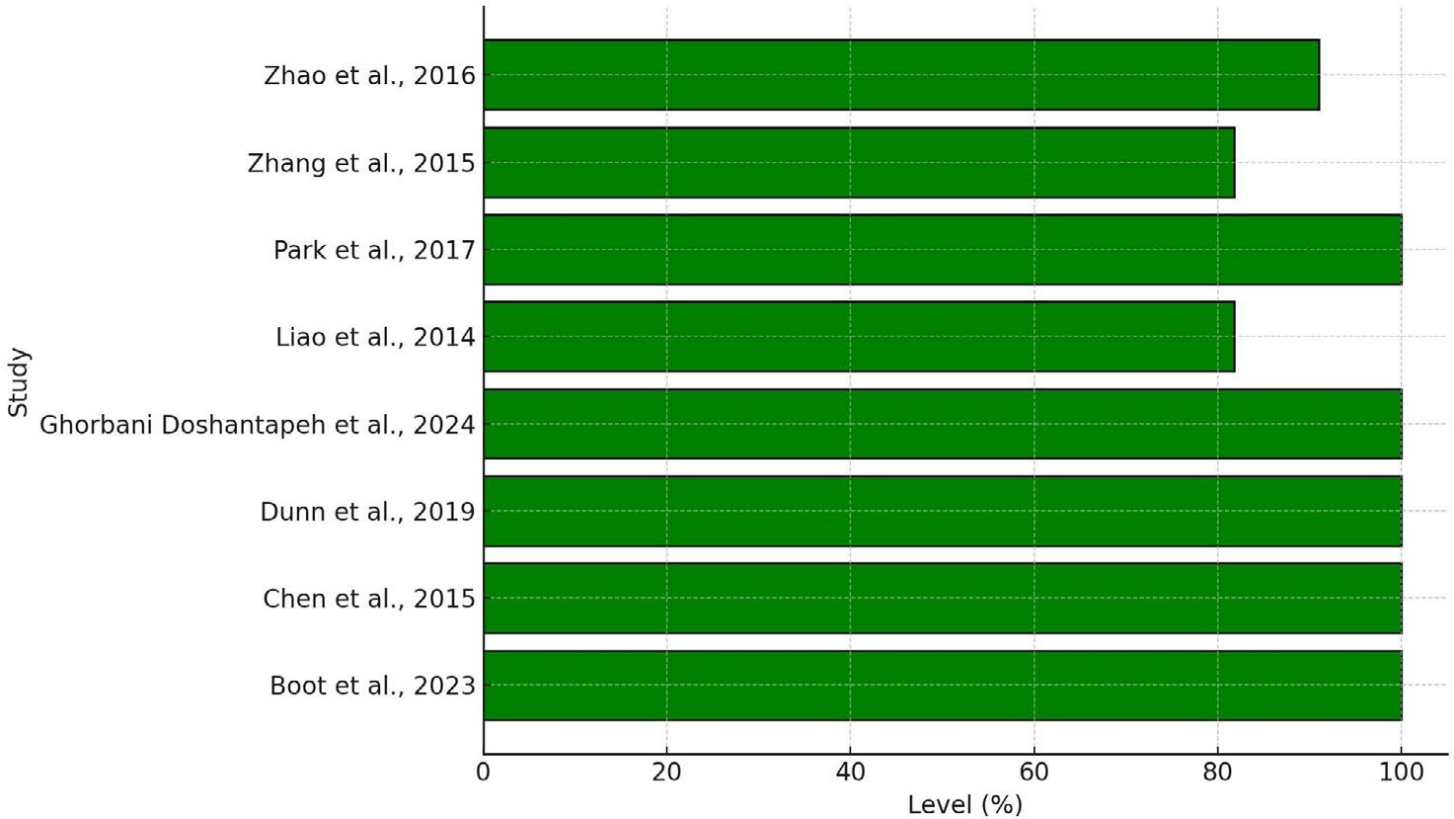
Quality and bias assessment. Quality/Bias according to JBI critical appraisal tools and Sguanci et al. [33].

All the studies included in this umbrella review demonstrated adequate methodological quality and a low risk of bias, with an average score of 94.3% (range: 81.8%–100%) (Figure 2).

### 3.3 Assessment of Overlap of the Included Studies

The analysis of potential overlap among the primary studies included in the systematic reviews revealed a very high overlap (CCA = 15.5%). To help clarify the sources of overlap or divergence, exposure types and outcomes have been added for each included study in the Supporting Information (Table S4).

### 3.4 Characteristics of the Included Studies

The studies included in this umbrella review [37, 38, 39, 40, 41, 42, 43, 44] show a geographically diverse distribution, with a predominant focus on Western countries, accounting for six studies (87.5% of the total), including the United States, Finland, Spain, Denmark, the Netherlands, United Kindom, and Belgium [37, 38, 39, 41, 43, 44]. Additional contributions came from Iran [40], and South Korea [42], each represented by a single study (12.5%). The main characteristics of the included studies are summarized in Table 1.

**TABLE 1:** Characteristics of the Included Studies.

Author, year, country	Study design	Studies included	Sample	Objective	Results	Quality assessment
Boot et al. (2023), Netherlands [37]	Pooled analysis (Prospective Cohort)	10	*N* = 519.996	Assess the relation between vitamin D intake and bladder cancer risk	High vitamin D intake with moderate calcium and low phosphorus reduces bladder cancer risk	JBI
Chen et al. (2015), USA, Finland, Spain, and Denmark, Netherlands [38]	Meta‐Analysis (Case–Control and Cohort)	17	*N* = 13,885 (IG)	Investigate the association of vitamins C, D, and E with bladder cancer risk	Circulating vitamin D and α‐tocopherol protective; no effect for vitamin C	JBI
Dunn et al. (2019), UK [39]	Systematic review	6	*N* = 536,141	Analyze the association between serum 25(OH)D levels and bladder cancer risk	Low serum 25(OH)D linked to higher bladder cancer risk; vitamin D supports immune response	JBI
Ghorbani Doshantapeh et al. (2024), Iran [40]	SR and meta‐analysis (Case–Control, Cohort, RCTs)	13	*N* = 528,259	Examine vitamin D levels and bladder cancer risk	Vitamin D < 50 nmol/L increases bladder cancer risk; ≥ 50 nmol/L protective (ages 40–49); no effect > 50	JBI
Liao et al. (2014), USA, Finland, Spain, and Denmark [41]	SR or meta‐analysis	5	*N* = 89,610	Assess impact of 25‐hydroxyvitamin D on bladder cancer risk	High 25(OH)D reduces bladder cancer risk (RR = 0.75, 95% CI: 0.65–0.87, *p* < 0.001)	Newcastle–Ottawa Scale
Park et al. (2017), South Korea [42]	Meta‐analysis of RCTs	14	*N* = 147,383	Evaluate effectiveness of vitamins/antioxidant supplements in bladder cancer prevention	No significant association with bladder cancer risk (RR = 1.04, 95% CI: 0.92–1.17)	Jadad Scale
Zhang et al. (2015), USA, Finland, Spain, Denmark, Netherlands [43]	Meta‐analysis	7	*N* = 62,141	Explore vitamin D status and bladder cancer risk	Vitamin D deficiency associated with increased bladder cancer risk (RR = 1.34, 95% CI: 1.17–1.53)	Newcastle–Ottawa Scale
Zhao et al. (2016), USA, Finland, Belgium, Spain, Denmark [44]	SR and network meta‐analysis	7	*N* = 90,757	Compare serum 25(OH)D concentrations and bladder cancer risk	Serum 25(OH)D ≥ 74 nmol/L reduces bladder cancer risk by 60%	Newcastle–Ottawa Scale

*Note:* Jadad scale: A scale used to assess the quality of randomized controlled trials, where a score of 3 or more indicates high quality; Newcastle‐Ottawa scale: A tool for assessing the quality of non‐randomized studies, with a higher score indicating higher study quality (6+ stars indicate high quality).

Abbreviations: 25(OH)D, 25‐hydroxyvitamin D; CG, control group; CI, confidence interval; IG, intervention group; JBI, Joanna Briggs Institute; LUTS, lower urinary tract symptoms; RCT, randomized controlled trial; RR, relative risk; SR, systematic review.

The majority of the studies focused on assessing the association between serum vitamin D levels and bladder cancer risk [38, 39, 40, 41, 42, 43, 44]. Additionally, several studies examined the relationship between vitamin D intake and bladder cancer risk [37, 38, 43]. These findings are summarized in Table 2.

**TABLE 2:** Vitamin D and bladder cancer risk.

Author, year, country	Outcomes	Vitamin D protective level/study findings	Nutritional intake (prevention bladder cancer)	Estimated effects	Heterogeneity (*I* ^2^)	*p*	Quality
Boot et al. (2023), Netherlands [37]	Dietary vitamin D intake and bladder cancer risk	High vitamin D intake combined with moderate calcium and low phosphorus intake reduces bladder cancer risk	Vitamin D intake: 0.00056 μg/cal (IG); 0.00042 μg/calorie (CG)	HR = 0.77, (95% CI: 0.59–1.00)	NR	< 0.001	Low
Chen et al. (2015), USA, Finland, Spain, and Denmark, Netherlands [38]	Vitamin D from diet and supplements^a^ Circulating serum vitamin D^b^	Increase in serum vitamin D level every 10 nmol/L: Associated with a 5% reduction in bladder cancer risk^b^	Increase in dietary vitamin D intake every 100 IU/day: No significant association with bladder cancer risk^a^	RR^a^ = 0.99 (95% CI: 0.95–1.03) RR^b^: 0.95 (95% CI: 0.90–1.00)	*I* ^2^ = 51.7%	0.78^a^ 0.10^b^	Low
Dunn et al. (2019), UK [39]	Serum Vitamin D Level and bladder cancer risk	Serum 25(OH)D ≥ 30–50 nmol/L associated with lower bladder cancer risk	NA	NR	NR	NR	Low
Ghorbani Doshantapeh et al. (2024), Iran [40]	Serum Vitamin D Level and bladder cancer risk	Vitamin D ≤ 50 nmol/L increases bladder cancer risk	NA	OR = 1.33 (95% CI 1.08, 1.64)	*I* ^2^ = 84.3%–91.3%	< 0.05	Low
Liao et al. (2014), USA, Finland, Spain, and Denmark [41]	Serum Vitamin D Level and bladder cancer risk	High serum 25‐hydroxyvitamin D level associated with 25% reduced risk of bladder cancer	NA	RR = 0.75 (95% CI: 0.65–0.87)	*I* ^2^ = 0%	≤ 0.001	Low
Park et al. (2017), Korea [42]	Serum Vitamin D Level and bladder cancer risk	Vitamin D supplements show no protective effect against bladder cancer	NR	RR = 1.05 (95% CI 0.85–1.29)	NA	NR	Low
Zhang et al. (2015), USA, Finland, Spain, Denmark, Netherlands [43]	Serum vitamin D level^c^ and dietary intake^d^ related to bladder cancer risk	Low serum vitamin D associated with increased bladder cancer risk	NA	RR = 1.32 (95% CI: 1.15–1.52)^c^ RR = 1.45, (95% CI: 0.99–2.13)^d^	*I* ^2^ = 0%^c,d^	0.001^c^ 0.005^d^	Low
Zhao et al. (2016), USA, Finland, Belgium, Spain, Denmanrk [44]	Serum Vitamin D Level and bladder cancer risk	Protective level: Serum 25(OH)D ≥ 74–75 nmol/L: ≥ 75 nmol/L^e^; ≥ 74 nmol/L^f^	NA	OR 0.68 (95% CI: 0.52–0.87)^e^ *R* ^2^ = 0.98^f^	NR	0.007^e^	Low

*Note:* The quality of the studies was assessed using the JBI Critical Appraisal Tool. Scores > 70% were classified as high quality (+++), scores between 50% and 70% as medium quality (++), and scores < 50% as low quality (+). (^a–f^) Different outcomes or subgroup analyses within the same study and correspond to the specific results reported in the table. Quality/Bias according to JBI critical appraisal tools and Sguanci et al. [33].

Abbreviations: CG, control group; IG, intervention group; LUTS, lower urinary tract symptoms; NA, not applicable; NR, not reported.

### 3.5 Vitamin D Serum Levels and Bladder Cancer Risk

In line with the definitions used across the included studies, “adequate” serum vitamin D was considered as ≥ 30 nmol/L, and “deficiency” as < 30 nmol/L.

Several studies have explored the link between vitamin D levels and the risk of bladder cancer, suggesting a protective role for this vitamin in preventing the disease. A Meta‐analysis [40] reports that individuals with serum 25‐hydroxyvitamin D levels below 50 nmol/L have a significantly higher bladder cancer risk: +33% overall, +87% for non‐muscle‐invasive bladder cancer (NMIBC), and 1.7 times for muscle‐invasive bladder cancer (MIBC). This protective effect is age‐dependent, showing a 49% risk reduction in those aged 40–49 with levels ≥ 50 nmol/L, but no significant effect in those over 50, indicating that vitamin D deficiency may be an independent bladder cancer risk factor. Another study [38] showed a decreasing bladder cancer risk with every 10 nmol/L increase in vitamin D (RR = 0.95; 95% CI: [0.90–1.00]), though this was not statistically significant (*p* = 0.10).

Two meta‐analyses further confirmed the protective role of serum vitamin D: Liao et al. [41] with five studies found a 25% risk reduction (RR = 0.75; 95% CI: [0.65–0.87]; *p* < 0.001) for those with higher vitamin D levels, while Zhang et al. [43] with seven studies reported a 34% increased risk (RR = 1.34; 95% CI: [1.17–1.53]; *p* < 0.0001) for bladder cancer in individuals with lower vitamin D levels, highlighting the increased risk associated with vitamin D deficiency [42]. By aggregating the results of previous meta‐analyses into a new fixed‐effect meta‐analysis, an overall protective and statistically significant effect is observed (RR = 0.92, 95% CI [0.88; 0.97], *p* = 0.0006). However, the high heterogeneity among studies (*I* ^2^ = 81%, τ^2^ = 0.0226, *p* < 0.01) makes a random‐effects model more appropriate, which yields a non‐statistically significant effect (RR = 0.86, 95% CI [0.63; 1.16], *p* = 0.2025). Although the result suggests a possible risk reduction (RR = 0.86), the substantial variability across studies introduces uncertainty and prevents definitive conclusions in adequate serum vitamin D studies, as shown in Table 3.

**TABLE 3:** Adequate serum vitamin D level and bladder cancer risk.

Study	RR	95% CI	%W (common effects model)	%W (random effects model)
Chen et al. (2015)	0.95	[0.90; 1.00]	80.90%	32.20%
Liao et al. (2014)	0.75	[0.65; 0.87]	10.60%	26.70%
Zhao et al. (2016)	0.68	[0.53; 0.88]	3.40%	18.90%
Park et al. (2017)	1.05	[0.85; 1.29]	5.20%	22.20%

*Note:* Adequate serum vitamin D was defined as ≥ 30 nmol/L; deficiency as < 30 nmol/L. Referent group: patients with low serum vitamin D levels (< 30 nmol/L).

Abbreviations: CI, confidence interval; RR, relative risk; W, weight.

**FIGURE 3:**
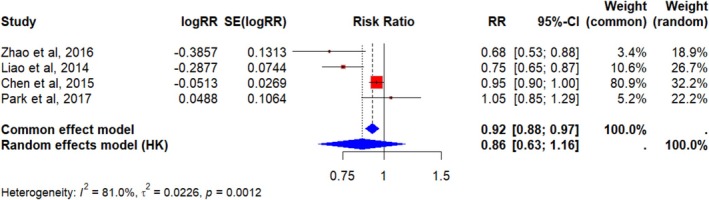
Adequate serum vitamin D levels and bladder cancer risk—meta‐analysis. Forest plot of the Adequate Serum Vitamin D Level studies meta‐analysis: Each study is represented by a red square proportional to its weight; horizontal bars indicate the 95% CI. The blue diamond shows the overall effect estimate with its 95% CI. The vertical line indicates the null effect (RR = 1). Adequate serum vitamin D was defined as ≥ 30 nmol/L; deficiency as < 30 nmol/L.

The results of the meta‐analysis on adequate serum vitamin D levels are shown in Figure 3.

A second meta‐analysis was conducted on the two reviews reporting data on the association between low serum vitamin D levels and bladder cancer risk. This analysis showed a 32% increased risk, statistically significant under both models (fixed effects: *p* < 0.0001; random effects: *p* = 0.0079). The absence of heterogeneity and the high consistency between the two reviews suggest that, despite being based on only two studies [40, 43], the findings are robust. However, the overlap analysis indicated that five primary studies were shared, while two were unique to Zhang [43] and eight to Ghorbani [40], resulting in a CCA of 33.3% (very high overlap), as shown in Table 4.

**TABLE 4:** Low serum vitamin D levels and bladder cancer risk.

Study	RR	95% CI	%W (common effects model)	%W (random effects model)
Ghorbani et al. (2024)	1.33	[1.08; 1.64]	30.80%	30.80%
Zhang et al. (2015)	1.32	[1.15; 1.52]	69.20%	69.20%

*Note:* Low serum vitamin D was defined as < 30 nmol/L. Referent group: patients with adequate serum vitamin D levels (≥ 30 nmol/L).

Abbreviations: CI, confidence interval; RR, relative risk; W, weight.

**FIGURE 4:**
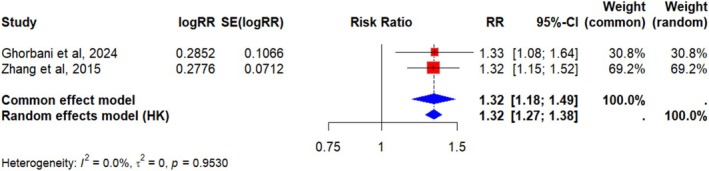
Low serum vitamin D levels and bladder cancer risk—meta‐analysis. Forest plot of the meta‐analysis: Each study is represented by a red square proportional to its weight; horizontal bars indicate the 95% CI. The blue diamond shows the overall effect estimate with its 95% CI. The vertical line indicates the null effect (RR = 1). Adequate serum vitamin D was defined as ≥ 30 nmol/L; deficiency as < 30 nmol/L.

The results of the meta‐analysis on low serum vitamin D levels are shown in Figure 4.

### 3.6 Vitamin D Nutritional Intake and Bladder Cancer Risk

The study by Boot et al. [37], which analyzed a large cohort of 1994 bladder cancer cases and 518,002 controls, found no significant direct association between dietary vitamin D intake and bladder cancer risk. However, a combination of high vitamin D intake with moderate calcium and low phosphorus intake was associated with a 23% reduced bladder cancer risk (HR = 0.77; 95% CI: 0.59–1.00). Subgroup analyses by age, gender, and region showed consistent results, with no significant risk reduction from higher dietary vitamin D alone. A slight, non‐significant protective trend was seen in some groups, like females [37]. Further analysis by Chen et al. [38] and other studies included in meta‐analyses highlighted that individuals with lower dietary vitamin D intake had higher bladder cancer incidence. Bladder cancer patients consumed on average 100–300 IU/day, compared to controls who consumed 200–400 IU/day. Some meta‐analyses suggested a potential risk reduction with higher dietary vitamin D intake (HR < 1.0), but findings varied across studies [38]. Finally, a meta‐analysis [43] combining multiple studies found a modest association between low dietary vitamin D intake and increased bladder cancer risk (RR = 1.45; 95% CI: [0.99–2.13]; *p* = 0.05).

## 4 Discussion

Bladder Cancer presents a significant global health challenge. This umbrella review examined the relationship between serum vitamin D levels, dietary intake, and supplementation with bladder cancer risk. Overall, the findings indicate that low serum vitamin D (< 50 nmol/L) is consistently associated with increased bladder cancer risk, whereas higher levels (> 75 nmol/L) may confer protection [38, 39, 41, 44]. For instance, Zhao et al. [44] reported a 32% reduction in risk for individuals with serum levels above 75 nmol/L, and Liao et al. [41] observed a 25% reduction in bladder cancer risk in individuals with high serum vitamin D levels.

Beyond these epidemiological findings, biological mechanisms may partly explain the observed associations: vitamin D's active form (calcitriol) binds to the vitamin D receptor (VDR) in bladder tissue, regulating cell growth, differentiation, and apoptosis. In addition, vitamin D modulates immune responses and reduces chronic inflammation, both processes relevant to tumor progression [39].

Daily dietary intake alone showed no strong or direct association with bladder cancer risk [37, 42]. This suggests that vitamin D's bioavailability and metabolism, influenced by factors such as sun exposure and receptor variability, are more critical than intake itself. Zhao et al. [44], through a network meta‐analysis of seven studies, confirmed that serum levels above 75 nmol/L were more protective, while Park et al. [42] also found no significant effect of vitamin or antioxidant supplements in 14 RCTs, reinforcing that intake or supplementation alone does not consistently reduce bladder cancer risk.

Genetic variability and the role of the vitamin D receptor (VDR) may partly explain inconsistencies, but extended details on specific polymorphisms or vitamin D binding protein (DBP) have been condensed [45, 46]. The distinction between total and free vitamin D, and the role of DBP may be relevant for bioavailability and warrants further investigation [46].

Some degree of heterogeneity was reported across studies: moderate in Chen et al. (*I* ^2^ = 51.7%) [38], substantial in Dunn et al. (84.3%–91.3%) [39], while others such as Liao et al. [41] and Zhang et al. [43] reported none (*I* ^2^ = 0%), and Park et al. [42] and Zhao et al. [44] did not report values. This variability, together with the study overlap (CCA = 15.5%), suggests that results should be interpreted with caution.

Ultimately, the relationship between dietary vitamin D intake and bladder cancer remains debated. Boot et al. [37] found no significant correlation between intake and bladder cancer, whereas other reviews suggested a modest protective effect when intake was combined with certain nutritional factors [38, 43]. These discrepancies may depend on population‐specific diet and sun exposure.

Future research should adopt standardized methods for vitamin D measurement, clarify the role of free versus total vitamin D [47, 48], and account for population‐specific and genetic factors [49]. Well‐designed prospective studies and clinical trials are needed to confirm whether adequate vitamin D truly reduces bladder cancer risk. The broad scientific interest in vitamin D, also investigated in other clinical contexts such as hematopoietic stem cell transplantation [46], further underlines the importance of standardized approaches and mechanistic studies to better define its role in cancer prevention.

Lastly, this umbrella review highlights a consistent association between low serum vitamin D and increased bladder cancer risk, while evidence on intake and supplementation remains inconclusive. The discussion has been streamlined to focus on prevention and risk, in line with the scope of the review.

### 4.1 Limitations

Although the studies analyzed suggest that adequate levels of vitamin D may reduce the risk of bladder cancer, several limitations must be considered: methodological differences in measurement techniques and the variables assessed highlight the complexity of this relationship. For instance, the distinction between total and free vitamin D is rarely addressed in the included studies, despite its potential impact on biological activity. Using total serum vitamin D as a proxy may not fully capture its physiological effects, as only the free, unbound fraction is considered bioavailable and biologically active. This represents a relevant limitation when interpreting associations between vitamin D status and bladder cancer risk.

Moreover, individual and nutritional factors highlight the need for further research to determine which forms and levels of vitamin D are most relevant for prevention. Furthermore, understanding the interplay between biological and environmental factors remains crucial to elucidating the protective role of vitamin D.

A notable limitation of this review is the 15.5% overall overlap between the results of the included studies, indicating that several were based on similar primary data sources (e.g., large population‐based cohorts or registries). While this overlap may contribute to consistent findings across studies, it also necessitates caution when interpreting the results, as shared data sources may limit the independence of the evidence base. We have clarified this point to avoid confusion with the principle that meta‐analyses should include all relevant studies. Because the second meta‐analysis was based on only two reviews, meaningful sensitivity analyses could not be performed.

Moreover, it is important to acknowledge the inherent limitations of umbrella reviews themselves. These types of reviews rely on secondary data and often include systematic reviews and meta‐analyses with heterogeneous study designs, populations, and outcome measures. This diversity can pose challenges in synthesizing results and may affect the consistency and generalizability of the conclusions. As such, findings should be interpreted with caution, and future research should aim to validate these associations through primary data analysis and well‐controlled prospective studies.

## 5 Conclusion

The current body of evidence suggests that low serum vitamin D is consistently associated with an increased risk of bladder cancer, whereas higher levels may confer some protection. Maintaining serum vitamin D levels above 30 nmol/L may help reduce bladder cancer risk, although this threshold requires further validation. Evidence on dietary intake and supplementation remains inconclusive. The relationship between vitamin D and bladder cancer risk is complex, likely influenced by genetic, metabolic, and environmental factors. While promising, these findings require confirmation through well‐designed studies before vitamin D can be firmly established as a preventive strategy in bladder cancer.

## Author Contributions

Stefano Mancin: conceptualization, methodology, writing original draft, review and editing, investigation, visualization. Gaetano Ferrara: methodology, review and editing, investigation, visualization. Sara Morales Palomares: writing original draft, review and editing, investigation. Sofia Matteucci: review and editing, investigation, visualization. Alice Maria Santagostino: review and editing, investigation, visualization. Gaetano Ferrara: review and editing, investigation, visualization. Mauro Parozzi: review and editing, investigation, data analysis, validation. Fabio Petrelli: review and editing, visualization. Rodolfo Hurle: review and editing, validation, coordinator. Marco Sguanci: review and editing, investigation, visualization, coordinator. Giovanni Cangelosi: review and editing, investigation, visualization, coordinator.

## Funding

The publication fee for this work was covered by the Italian Ministry of Health’s 'Ricerca Corrente ' funding to IRCCS Humanitas Research Hospital.

## Conflicts of Interest

The authors declare no conflicts of interest.

## Supporting information


**Table S1:** Search strategy.
**Table S2:** JBI Critical appraisal tool for systematic review and research syntheses.
**Table S3:** Assessment of Overlap of the studies included.
**Table S4:** Overlap of the studies included.

## Data Availability

The datasets generated and/or analyzed during the current study are not publicly available due to ethical and privacy restrictions but are available from the corresponding author on reasonable request.
