# Supplementary material for: Vitamin D and Bladder Cancer Risk: An Umbrella Review and Second Order Meta‐Analysis
Source: Cancer Med. 2026 Mar 11;15(3):e71672. doi: 10.1002/cam4.71672 (PMC13093333; doi:10.1002/cam4.71672)
Supplement: Supplementary file 1 — Table S1: Search strategy. Table S2: JBI Critical appraisal tool for systematic review and research syntheses. Table S3: Assessment of Overlap of the studies included. Table S4: Overlap of the studies included. [file CAM4-15-e71672-s001.docx]

**Table S1 .** Search strategy

| **ID** | **Pubmed** | **Results** |
| --- | --- | --- |
| #1 | "bladder cancer" OR "urothelial carcinoma" OR "transitional cell carcinoma" OR "non-muscle invasive bladder cancer" OR "muscle invasive bladder cancer" OR "superficial bladder cancer" OR "invasive bladder cancer" OR "metastatic bladder cancer" OR "localized bladder cancer" OR "bladder tumor" OR "vesical neoplasm" |  |
| #2 | "Vitamin D"[Mesh] OR "Vitamin D" OR "Cholecalciferol" OR "Ergocalciferol" OR "D2" OR "D3" OR "Calciferol" OR "Calciol" OR "25-Hydroxyvitamin D" OR "25(OH)D" OR "1,25-Dihydroxyvitamin D" OR "1,25(OH)2D" OR "Secosteroid" OR "Vitamin D2" OR "Vitamin D3" |  |
| **#3** | **#1 AND #2**  **("Urinary Bladder Neoplasms"[Mesh] OR "Non-Muscle Invasive Bladder Neoplasms"[Mesh] OR "bladder cancer" OR "urothelial carcinoma" OR "transitional cell carcinoma" OR "non-muscle invasive bladder cancer" OR "muscle invasive bladder cancer" OR "superficial bladder cancer" OR "invasive bladder cancer" OR "metastatic bladder cancer" OR "localized bladder cancer" OR "bladder tumor" OR "vesical neoplasm") AND ("Vitamin D"[Mesh] OR "Vitamin D" OR "Cholecalciferol" OR "Ergocalciferol" OR "D2" OR "D3" OR "Calciferol" OR "Calciol" OR "25-Hydroxyvitamin D" OR "25(OH)D" OR "1,25-Dihydroxyvitamin D" OR "1,25(OH)2D" OR "Secosteroid" OR "Vitamin D2" OR "Vitamin D3")** | **230** |
| **ID** | **Cumulative Index Of Nursing And Allied Health Literature (CINAHL)** | **Results** |
| #1 | "bladder cancer" OR "urothelial carcinoma" OR "transitional cell carcinoma" OR "non-muscle invasive bladder cancer" OR "muscle invasive bladder cancer" OR "superficial bladder cancer" OR "invasive bladder cancer" OR "metastatic bladder cancer" OR "localized bladder cancer" OR "bladder tumor" OR "vesical neoplasm" |  |
| #2 | "Vitamin D” OR "Vitamin D" OR "Cholecalciferol" OR "Ergocalciferol" OR "D2" OR "D3" OR "Calciferol" OR "Calciol" OR "25-Hydroxyvitamin D" OR "25(OH)D" OR "1,25-Dihydroxyvitamin D" OR "1,25(OH)2D" OR "Secosteroid" OR "Vitamin D2" OR "Vitamin D3" |  |
| **#3** | **#1 AND #2**  **("Urinary Bladder Neoplasms"[Mesh] OR "Non-Muscle Invasive Bladder Neoplasms"[Mesh] OR "bladder cancer" OR "urothelial carcinoma" OR "transitional cell carcinoma" OR "non-muscle invasive bladder cancer" OR "muscle invasive bladder cancer" OR "superficial bladder cancer" OR "invasive bladder cancer" OR "metastatic bladder cancer" OR "localized bladder cancer" OR "bladder tumor" OR "vesical neoplasm") AND ("Vitamin D"[Mesh] OR "Vitamin D" OR "Cholecalciferol" OR "Ergocalciferol" OR "D2" OR "D3" OR "Calciferol" OR "Calciol" OR "25-Hydroxyvitamin D" OR "25(OH)D" OR "1,25-Dihydroxyvitamin D" OR "1,25(OH)2D" OR "Secosteroid" OR "Vitamin D2" OR "Vitamin D3")** | **25** |
| **ID** | **EMBASE** |  |
| #1 | "bladder cancer" OR "urothelial carcinoma" OR "transitional cell carcinoma" OR "non-muscle invasive bladder cancer" OR "muscle invasive bladder cancer" OR "superficial bladder cancer" OR "invasive bladder cancer" OR "metastatic bladder cancer" OR "localized bladder cancer" OR "bladder tumor" OR "vesical neoplasm" |  |
| #2 | "Vitamin D” OR "Vitamin D" OR "Cholecalciferol" OR "Ergocalciferol" OR "D2" OR "D3" OR "Calciferol" OR "Calciol" OR "25-Hydroxyvitamin D" OR "25(OH)D" OR "1,25-Dihydroxyvitamin D" OR "1,25(OH)2D" OR "Secosteroid" OR "Vitamin D2" OR "Vitamin D3" |  |
| **#3** | **#1 AND #2**  **('bladder cancer' OR 'urothelial carcinoma' OR 'transitional cell carcinoma' OR 'non-muscle invasive bladder cancer' OR 'muscle invasive bladder cancer' OR 'superficial bladder cancer' OR 'invasive bladder cancer' OR 'metastatic bladder cancer' OR 'localized bladder cancer' OR 'bladder tumor' OR 'vesical neoplasm') AND ('vitamin d' OR 'cholecalciferol' OR 'ergocalciferol' OR 'd2' OR 'd3' OR 'calciferol' OR 'calciol' OR '25-hydroxyvitamin d' OR '25(oh)d' OR '1,25-dihydroxyvitamin d' OR '1,25(oh)2d' OR 'secosteroid' OR 'vitamin d2' OR 'vitamin d3')** | **693** |
| **ID** | **COCHRANE LIBRARY** |  |
| #1 | "bladder cancer" OR "urothelial carcinoma" OR "transitional cell carcinoma" OR "non-muscle invasive bladder cancer" OR "muscle invasive bladder cancer" OR "superficial bladder cancer" OR "invasive bladder cancer" OR "metastatic bladder cancer" OR "localized bladder cancer" OR "bladder tumor" OR "vesical neoplasm" |  |
| #2 | "Vitamin D"[Mesh] OR "Vitamin D" OR "Cholecalciferol" OR "Ergocalciferol" OR "D2" OR "D3" OR "Calciferol" OR "Calciol" OR "25-Hydroxyvitamin D" OR "25(OH)D" OR "1,25-Dihydroxyvitamin D" OR "1,25(OH)2D" OR "Secosteroid" OR "Vitamin D2" OR "Vitamin D3" |  |
| **#3** | **#1 AND #2**  **"bladder cancer" OR "urothelial carcinoma" OR "transitional cell carcinoma" OR "non-muscle invasive bladder cancer" OR "muscle invasive bladder cancer" OR "superficial bladder cancer" OR "invasive bladder cancer" OR "metastatic bladder cancer" OR "localized bladder cancer" OR "bladder tumor" OR "vesical neoplasm" in Title Abstract Keyword AND "Vitamin D" OR "Vitamin D" OR "Cholecalciferol" OR "Ergocalciferol" OR "D2" OR "D3" OR "Calciferol" OR "Calciol" OR "25-Hydroxyvitamin D" OR "25(OH)D" OR "1,25-Dihydroxyvitamin D" OR "1,25(OH)2D" OR "Secosteroid" OR "Vitamin D2" OR "Vitamin D3" in Title Abstract Keyword - (Word variations have been searched)** | **Trials (n=17)** |

**TOTAL (n=965)**

**Table S2.** JBI Critical appraisal tool for systematic review and research syntheses

| **STUDY** | **ITEM 1** | **ITEM 2** | **ITEM 3** | **ITEM 4** | **ITEM 5** | **ITEM 6** | **ITEM 7** | **ITEM 8** | **ITEM 9** | **ITEM 10** | **ITEM 11** | **INCLUDE** | **EXCLUDE** | **LEVEL (%)** | **COMMENTS** |
| --- | --- | --- | --- | --- | --- | --- | --- | --- | --- | --- | --- | --- | --- | --- | --- |
| Boot et al., 2023, | Y | Y | Y | Y | Y | Y | Y | Y | Y | Y | Y | X |  | 100 |  |
| Chen et al., 2015, | Y | Y | Y | Y | Y | Y | Y | Y | Y | Y | Y | X |  | 100 |  |
| Dunn et al., 2019 | Y | Y | Y | Y | Y | Y | Y | Y | Y | Y | Y | X |  | 100 |  |
| Ghorbani Doshantapeh et al.. 2024, | Y | Y | Y | Y | Y | Y | Y | Y | Y | Y | Y | X |  | 100 |  |
| Liao et al., 2014, | Y | Y | Y | Y | Y | Y | Y | Y | Y | NA | NA | X |  | 81.8 |  |
| Park et al., 2017 | Y | Y | Y | Y | Y | Y | Y | Y | Y | Y | Y | X |  | 100 |  |
| Zhang et al., 2015 | Y | Y | Y | Y | Y | Y | Y | NA | NA | Y | Y | X |  | 81.8 |  |
| Zhao et al., 2016 | Y | Y | Y | Y | Y | Y | Y | Y | NA | Y | Y | X |  | 91 |  |

***Legend****: Y=Yes; N=No; U=Unclear; NA=Not Applicable / Items from Critical appraisal tool for systematic review and research syntheses: 1=Is the review question clearly and explicitly stated?; 2=Were the inclusion criteria appropriate for the review question? 3=Was the search strategy appropriate?; 4=Were the sources and resources used to search for studies adequate? 5=Were the criteria for appraising studies appropriate?; 6=Was critical appraisal conducted by two or more reviewers independently? 7=Were there methods to minimize errors in data extraction?;8=Were the methods used to combine studies appropriate; 9=Was the likelihood of publication bias assessed?;10=Were recommendations for policy and/or practice supported by the reported data?;11=Were the specific directives for new research appropriate?*

**Table S3.** Assessment of Overlap of the studies included

| **Study** | **Records included** | **Expousure Type** |
| --- | --- | --- |
| Boot et al., 2023, Netherlands | Goossens ME, et al. International pooled study on diet and bladder cancer: the bladder cancer, epidemiology and nutritional determinants (BLEND) study: design and baseline characteristics. Arch Publ Health 2016;74(1):30.  White E, et al. VITamins and Lifestyle cohort study: study design and characteristics of supplement users. Am J Epidemiol 2004;159(1):83e93.  Riboli E, et al. European Prospective Investigation into Cancer and Nutrition (EPIC): study populations and data collection. Publ Health Nutr 2002;5(6b):1113e24.  Riboli E, Kaaks R. The EPIC project: rationale and study design. European prospective investigation into cancer and nutrition. Int J Epidemiol 1997;26(Suppl 1):S6e14 | Diet |
| Chen et al., 2015, China | Brinkman, M. T. et al. Minerals and vitamins and the risk of bladder cancer: results from the New Hampshire Study. Cancer Causes Control. 21, 2010; 609–619  Michaud, D. S. et al. Prospective study of dietary supplements, macronutrients, micronutrients, and risk of bladder cancer in US men. Am J Epidemiol. 2000; 152, 1145–1153  Afzal, S., Bojesen, S. E. & Nordestgaard, B. G. Low plasma 25-hydroxyvitamin D and risk of tobacco-related cancer. Clin Chem. 59, 2013;771–780  Mondul, A. M., Weinstein, S. J., et al. Serum vitamin D and risk of bladder cancer in the Prostate, Lung, Colorectal, and Ovarian (PLCO) Cancer Screening trial. Cancer Epidemiol Biomarkers Prev. 21, 2012; 1222–1225  Mondul, A. M., et al. Influence of vitamin D binding protein on the association between circulating vitamin D and risk of bladder cancer. Br J Cancer 107, 2012; 1589–1594  Amaral, A. F. et al. Plasma 25-hydroxyvitamin D(3) and bladder cancer risk according to tumor stage and FGFR3 status: a mechanism-based epidemiological study. J Natl Cancer Inst. 2012; 104, 1897–1904    Brinkman, M. T. et al. Dietary intake of micronutrients and the risk of developing bladder cancer: results from the Belgian case-control study on bladder cancer risk. Cancer Causes Control. 2011; 22, 469–478 | Serum 25(OH)D, Diet and Supplements |
| Dunn et al., 2019, UK | Ben Fradj M.K. et al. , Bladder Cancer is associated with low plasma 25-hydroxyvitamin d concentrations in tunisian population, Nutr. Cancer 68 (2) 2016 208–213 2016.  Amaral A.F., et al. Plasma 25-hydroxyvitamin D(3) and bladder cancer risk according to tumor stage and FGFR3 status: a mechanism-based epidemiological study, J. Natl. Cancer Inst. 104 (24) (2012) 1897–1904.  Mondul A.M, et al. Serum vitamin D and risk of bladder cancer, Cancer Res. 70 (22) (2010) 9218–9223.  Peiris A.N, et al. Relationship of vitamin D monitoring and status to bladder cancer survival in veterans, South. Med. J. 106 (2) (2013) 126–130.  Mondul A.M, et al. Serum vitamin D & risk of bladder cancer in the Prostate, Lung, Colorectal, and Ovarian (PLCO) Cancer screening trial, Cancer Epidemiol. Biomark. Prev. 21 (7) (2012) 1222–1225 | Serum 25(OH)D |
| Ghorbani Doshantapeh et al., 2024, Iran | Wu E, et al. Association of serum 25-hydroxyvitamin D with the  incidence of 16 cancers, cancer mortality, and all-cause  mortality among individuals with metabolic syndrome: a  prospective cohort study. Eur J Nutr. 2023;62:2581-92. doi:  10.1007/s00394-023-03169-x.  Abdelgawad A, et al.A prospective trial investigating the role of Serum  25-hydroxyvitamin D in diagnosis and prognosis of bladder  cancer. PLoS One. 2022;17:e0266371. doi: 10.1371/journal.  pone.0266371.  Hektoen HH, et al. Vitamin D and vitamin D-binding protein and risk of bladder cancer: a nested case control study in the Norwegian Janus Serum Bank Cohort. Cancer Med. 2021;10:4107-16. doi: 10.1002/cam4.3960.  Ben Fradj MK, et al. Bladder cancer is associated with low plasma 25-hydroxyvitamin D concentrations in Tunisian population. Nutr Cancer. 2016;68:208-13. doi: 10.1080/01635581.2016.1134598.  Afzal S, et al. Low plasma 25-hydroxyvitamin D and risk of tobacco-related cancer. Clin Chem. 2013;59:771-80. doi: 10.1373/  clinchem.2012.201939.  Amaral AF, et al. Plasma 25-hydroxyvitamin D3 and bladder cancer risk according to tumor stage and FGFR3 status: a mechanism-based epidemiological study. J Natl Cancer Inst. 2012;104:1897-904. doi: 10.1093/jnci/djs444.  Mondul AM, et al. Influence of vitamin D binding protein on the association between circulating vitamin D and risk of bladder cancer. Br J Cancer. 2012;107:1589-94. doi: 10.1038/bjc.2012.417. (2012a)  Mondul AM, et al. Serum vitamin D and risk of bladder cancer in the prostate, lung, colorectal, and ovarian (PLCO) cancer screening trial.  Cancer Epidemiol Biomarkers Prev. 2012;21:1222-5. doi:  10.1158/1055-9965.epi-12-0439. (2012b)  Mondul AM, et al. Serum vitamin D and risk of bladder  cancer. Cancer Res. 2010;70:9218-23. doi: 10.1158/0008-  5472.can-10-0985.  Boot IW, et al. Dietary vitamin D intake and the bladder cancer  risk: a pooled analysis of prospective cohort studies. Clin  Nutr. 2023;42:1462-74. doi: 10.1016/j.clnu.2023.05.010.  Brinkman MT, et al. Minerals and vitamins and the risk of bladder cancer: results from the New Hampshire Study. Cancer Causes Control. 2010;21:609-19. doi: 10.1007/s10552-009-9490-0  Brinkman MT, Buntinx F, Kellen E, Dagnelie PC, Van  Dongen MC, Muls E, et al. Dietary intake of micronutrients  12 Journal of Nephropharmacology, Volume 13, Issue 2, 2024 <https://jnephropharmacology.com>  Leung HW, et al. Vitamin D3 intake dose and common cancer: a population-based case control study in a Chinese population. J Cancer. 2016;7(14):2028-34. doi: 10.7150/jca.16505. | Serum 25(OH)D and Diet |
| Liao et al., 2014, China | Giovannucci E, et al. Prospective study of predictors of vitamin D status and cancer incidence and mortality in men. J Natl Cancer Inst. 2006;98:451–9.  Amaral AF, et al. Plasma 25-hydroxyvitamin D(3) and bladder cancer risk according to tumor stage and FGFR3 status: a mechanism-based epidemiological study. J Natl Cancer Inst. 2012;104:1897–904.  Afzal S, et al . Low plasma 25-hydroxyvitamin D and risk of tobacco-related cancer. Clin Chem.2013;59:771–80.  Mondul AM, et al. Serum vitamin D and risk of bladder cancer in the prostate, lung, colorectal, and ovarian (PLCO) cancer screening trial.  Cancer Epidemiol Biomarkers Prev. 2012;21:1222-5. doi:  10.1158/1055-9965.epi-12-0439.  Mondul AM, et al. Influence of vitamin D binding protein on the association between circulating vitamin D and risk of bladder cancer. Br J Cancer. 2012;107:1589–94. | Serum 25(OH)D |
| Park et al., 2017, Korea | Nepple KG, et al. Bladder Cancer Genitourinary Oncology Study Group. Bacillus Calmette-Guérin with or without interferon α-2b and megadose versus recommended daily allowance vitamins during induction and maintenance intravesical treatment of nonmuscle invasive bladder cancer. J Urol 2010; 184: 1915-9. | Supplement (antioxidants/vitamins) |
| Zhang et al., 2015, China | Giovannucci E, et al. Prospective study of predictors of vitamin d status and cancer incidence and mortality in men. J Natl Cancer Inst 2006;98:451-459.  Mondul AM, et al. Serum vitamin d and risk of bladder cancer. Cancer Res 2010;70:9218-9223.  Amaral AF, et al. Plasma 25-hydroxyvitamin d(3) and bladder cancer risk according to tumor stage and fgfr3 status: A mechanism-based epidemiological study. J Natl Cancer Inst 2012;104:1897-1904.  Afzal S, et al. Low plasma 25-hydroxyvitamin d and risk of tobacco-related cancer. Clin Chem 2013;59:771-780.  Brinkman MT, et al. Minerals and vitamins and the risk  of bladder cancer: Results from the new hampshire study. Cancer Causes Control 2010;21:609-619.  Brinkman MT, et al. Dietary intake of micronutrients and the risk of developing bladder cancer: Results from the belgian case-control study on bladder cancer risk. Cancer Causes Control 2011;22:469-478.  Mondul AM, et al. Serum vitamin D and risk of bladder cancer in the prostate, lung, colorectal, and ovarian (PLCO) cancer screening trial.  Cancer Epidemiol Biomarkers Prev. 2012;21:1222-5. doi:  10.1158/1055-9965.epi-12-0439. | Serum 25(OH)D |
| Zhao et al., 2016, China | Afzal S, et al. Low plasma 25-hydroxyvitamin D  and risk of tobacco-related cancer. Clin Chem 2013;59:771–80.  Amaral AF, et al. Plasma 25-hydroxyvitamin D(3) and bladder cancer riskaccording to tumor stage and FGFR3 status: A mechanism-based epidemiological study. J Natl Cancer Inst 2012;104:1897–904.  [Brinkman MT, ,et al. Dietary intake of micronutrients and the risk of developing bladder cancer: Results from the Belgian case-control study on bladder cancer risk.Cancer Causes Control 2011;22:469–78.  Brinkman MTet al. Minerals and vitamins and the risk of bladder cancer: Results from the New Hampshire Study. Cancer Causes Control 2010;21:609–19.  Giovannucci E, et al. Prospective study of predictors of vitamin D status and cancer incidence and mortality in men. J Natl Cancer Inst 2006;98:451–9.  Mondul AM, et al. Serum vitamin D and risk of bladder cancer in the Prostate, Lung, Colorectal, and Ovarian (PLCO) Cancer Screening trial. Cancer Epidemiol Biomarkers Prev 2012;21:1222–5.  Mondul et al. Serum vitamin D and risk of bladder cancer. Cancer Res 2010;70:9218–23 | Serum 25(OH)D |

**Table S4.** Overlap of the studies included

| **CCA=15.5%** | Boot et al., 2023, Netherlands  Diet – Bladder cancer incidence | Chen et al., 2015, China 25(OH)D, Diet and Supplements– Bladder cancer incidence | Dunn et al., 2019, UK  25(OH)D Incidence and staging (NMIBC vs MIBC) | Ghorbani Doshantapeh et al., 2024, Iran  25(OH)D and Diet - Bladder cancer incidence | Liao et al., 2014, China 25(OH)D– Bladder cancer incidence  (MA) | Park et al., 2017, Korea Supplement (antioxidants/vitamins)  Bladder cancer incidence (RCT) | Zhang et al., 2015, China 25(OH)D– Bladder cancer incidence | Zhao et al., 2016, China 25(OH)D - Bladder cancer incidence (network MA) |
| --- | --- | --- | --- | --- | --- | --- | --- | --- |
| Goossens ME (2016) |  |  |  |  |  |  |  |  |
| White E (2004) |  |  |  |  |  |  |  |  |
| Riboli E (2002) |  |  |  |  |  |  |  |  |
| Riboli E (1997) |  |  |  |  |  |  |  |  |
| Brinkman MT (2010) |  |  |  |  |  |  |  |  |
| Brinkman, MT (2011) |  |  |  |  |  |  |  |  |
| Michaud, DS (2000) |  |  |  |  |  |  |  |  |
| Afzal, S (2013) |  |  |  |  |  |  |  |  |
| Mondul, AM (2012a) |  |  |  |  |  |  |  |  |
| Mondul, AM (2012b) |  |  |  |  |  |  |  |  |
| Amaral, AF (2012) |  |  |  |  |  |  |  |  |
| Ben Fradj MK (2016) |  |  |  |  |  |  |  |  |
| Mondul AM (2010) |  |  |  |  |  |  |  |  |
| Peiris AN (2013) |  |  |  |  |  |  |  |  |
| Wu E (2023) |  |  |  |  |  |  |  |  |
| Abdelgawad A (2022) |  |  |  |  |  |  |  |  |
| Hektoen HH (2021) |  |  |  |  |  |  |  |  |
| Boot IW (2023) |  |  |  |  |  |  |  |  |
| Brinkman MT (2010) |  |  |  |  |  |  |  |  |
| Brinkman MT (2024) |  |  |  |  |  |  |  |  |
| Leung HW (2016) |  |  |  |  |  |  |  |  |
| Giovannucci E (2006) |  |  |  |  |  |  |  |  |
| Nepple KG (2010) |  |  |  |  |  |  |  |  |
| Brinkman MT (2011) |  |  |  |  |  |  |  |  |

***Legend****. (50-24)/(8*24)-24: 26/168 =* ***15.5% overlap;*** NMIBC*: Non-Muscle-Invasive Bladder Cancer;* MIBC*: Muscle-Invasive Bladder Cancer;* RCT*: Randomized Controlled Trial;* MA*: Meta-analysis;* 25(OH)D*: Serum 25-hydroxyvitamin D.*
